# Germline genetic biomarkers to stratify patients for personalized radiation treatment

**DOI:** 10.1186/s12967-022-03561-x

**Published:** 2022-08-12

**Authors:** Ida Deichaite, Austin Hopper, Lena Krockenberger, Timothy J. Sears, Leisa Sutton, Xenia Ray, Andrew Sharabi, Ami Navon, Parag Sanghvi, Hannah Carter, Vitali Moiseenko

**Affiliations:** 1 Department of Radiation Medicine and Applied Sciences, University of California San Diego, La Jolla, CA USA; 2 Moores Cancer Center, University of California San Diego, La Jolla, CA USA; 3 Bioinformatics and Systems Biology Program, University of California San Diego, La Jolla, CA USA; 4 Department of Biological Regulation, Weizmann Institute of Science, Rehovot, Israel; 5 Division of Medical Genetics, Department of Medicine, University of California San Diego, La Jolla, CA USA

**Keywords:** Predictive biomarkers of radiation toxicity, Head and neck squamous cell carcinoma, Radiogenomics, Germline variants, TSC2, HLA-A, TET2, GEN1, NCOR2

## Abstract

**Background:**

Precision medicine incorporating genetic profiling is becoming a standard of care in medical oncology. However, in the field of radiation oncology there is limited use of genetic profiling and the impact of germline genetic biomarkers on radiosensitivity, radioresistance, or patient outcomes after radiation therapy is poorly understood. In HNSCC, the toxicity associated with treatment can cause delays or early cessation which has been associated with worse outcomes. Identifying potential biomarkers which can help predict toxicity, as well as response to treatment, is of significant interest.

**Methods:**

Patients with HNSCC who received RT and underwent next generation sequencing of somatic tumor samples, transcriptome RNA-seq with matched normal tissue samples were included. Patients were then grouped by propensity towards increased late vs. early toxicity (Group A) and those without (Group B), assessed by CTCAE v5.0. The groups were then analyzed for association of specific germline variants with toxicity and clinical outcomes.

**Results:**

In this study we analyzed 37 patients for correlation between germline variants and toxicity. We observed that TSC2, HLA-A, TET2, GEN1, NCOR2 and other germline variants were significantly associated with long term toxicities. 34 HNSCC patients treated with curative intent were evaluated for clinical outcomes. Group A had significantly improved overall survival as well as improved rates of locoregional recurrence and metastatic disease. Specific variants associated with improved clinical outcomes included TSC2, FANCD2, and PPP1R15A, while the HLA-A and GEN1 variants were not correlated with survival or recurrence. A group of five HLA-DMA/HLA-DMB variants was only found in Group B and was associated with a higher risk of locoregional recurrence.

**Conclusions:**

This study indicates that germline genetic biomarkers may have utility in predicting toxicity and outcomes after radiation therapy and deserve further investigation in precision radiation medicine approaches.

## Background

Radiation therapy (RT) contributes to 40% of all cancer cures world-wide and improves the quality of life for many others [1]. Some patients tolerate treatments extremely well, but others experience severe RT adverse events (rtAEs) that can have lasting and debilitating characteristics and negatively impact patient quality of life. In the clinic, radiation doses for patients are currently prescribed in a “one-size-fits-all” approach and are independent of their genetics profile. Personalizing RT for each patient, based on a radiosensitivity profile determined from their individual genetics and the genetic characteristics of their cancer, could significantly improve cancer patients’ quality of life in the long term.

In this work we chose to study head and neck squamous cell carcinoma (HNSCC) as a model cancer type in which to identify genetic biomarkers that could be used to personalize a course of radiotherapy to improve patient outcomes and quality of life. In HNSCC, frequent rtAEs include dysphagia, xerostomia, and cutaneous fibrosis [2], which are debilitating to the patients who otherwise benefit from curative RT of their tumor. Significant technological advancements including intensity modulated RT (IMRT) have allowed for meaningful reductions in dose to uninvolved organs at risk (OAR) [3–5]. Despite these innovations, HNSCC RT toxicity continues to have a significant impact on patient recovery and quality of life, often resulting in delays or premature termination of treatment, which are both associated with higher rates of local recurrence [6–8]. Specifically, missing two or more treatments has been associated with increased recurrence risk and inferior overall survival (OS), with the decrement to OS estimated at 1% per 1 missed day [9]. Several reports including surveillance, epidemiology, and end results (SEER) analysis of more than 300,000 head and neck cancer (HNC) patients, have shown excessive rates of suicide in survivors of HNSCC, second only to survivors of pancreatic cancer [10, 11].

There is substantial literature implicating patient germline variants as factors in influencing patient-specific radiosensitivity. For example, deoxyribonucleic acid (DNA) repair genes, such as ATM serine/threonine kinase (ATM) germline variants, are known to have significant effects on radiosensitivity [12–14]. Other clinical conditions such as Nijmegen breakage syndrome, Fanconi anemia, retinoblastoma and Riddle syndrome are characterized for genetic variants that contribute to cellular and clinical radiosensitivity [15–21]. Similar associations of specific germline variants and toxicity outcomes have been described in prostate and non-small cell lung cancer (NSCLC) [22]. In a recent study an association with mucositis RT induced toxicity in HNSCC and a specific locus on chromosome 5 was reported [23]. Here, we proceed to analyze the association of germline variants with HNSCC rtAEs.

As a benchmark for assessment, in this exploratory study patients were evaluated based on their overall toxicity profiles as well as by assessing increase in late versus early toxicity symptoms. Here, we report the germline variants associated with RT toxicity in HNSCC.

## Methods

### Data source and patient inclusion criteria

This retrospective analysis was approved by our institutional review board (UCSD HRPP#200495). Thirty-seven HNSCC patients who underwent Tempus xT somatic tumor testing paired with normal matched specimens and received RT with available dosimetric data were selected for this study. All selected patients in our study cohort (n = 37) were treated at the Moores Cancer Center at the University of California San Diego between 2009 and 2021.

The CAP/CLIA validated Tempus xT test is ordered by a clinician to provide predictive, prognostic, and therapeutic management for patients. Patients were consented for the test in accordance with federal, state, University, and UCSD Human Research Protection Program policies. Normal matched specimen sources accepted for testing include blood or saliva, collected at time of ordering the test.

### Patient demographics and treatment variables

We acquired patient characteristics and radiation data for these 37 patients including age at diagnosis, gender, smoking history, and human papillomavirus (HPV) status. Treatment parameters including pre-RT surgical resection, radiation dose, and induction/concurrent systemic therapy were recorded as well. Staging information was collected according to the American Joint Committee on Cancer (AJCC) classification edition in effect at the time of diagnosis, ranging from 6 to 8th edition [24].

### Collection of toxicity data

Patient charts were utilized to report early and late rtAE endpoints for mucositis, dysphagia and xerostomia. Toxicities were recorded using Common Terminology Criteria for Adverse Events (CTCAE) v.5.0, which were scored and reported by the treating physician on the day of service during therapy and in follow up. Early toxicity endpoints were recorded as the highest CTCAE grade experienced during therapy or within 6 weeks of completing therapy. Late toxicity endpoints were recorded as the highest CTCAE grade experienced from 6 months post-RT to the time of most recent follow up.

### Statistical analysis

The relationship between categorical variables and each outcome was analyzed with a chi-square test where p-values ≤ 0.05 were significant. Differences in patient characteristics were compared using Fisher’s exact test or chi-square as appropriate. The effect size was calculated as the ratio of variant to wild type samples in group A divided by the ratio of variant to wild type samples in group B: (Mut A/WT A)/(Mut B/WT B). Differences in OS, locoregional recurrence (LRR), locoregional failure free survival (LFS), progression free survival (PFS), and metastasis free survival (MFS) were compared between Group A and Group B as well as Group C and group D by Kaplan–Meier (KM) survival analysis with log-rank testing for significance. Analysis was performed using SPSS V22.0 [25].

### RNA-seq analysis

Ribonucleic acid (RNA) sequencing (RNA-seq) FastQ files were processed for alignment and quality control. The reads were trimmed, and low-quality reads were removed using Trimgalore v 0.6.3_dev [26] with the “paired” parameter and length of 76 bps. Trimmed FastQ sequences were aligned to the human reference genome (GRCh38) using STAR aligner v2.7.1a. Bam files were sorted by coordinate by using option “–outSAMtype BAM SortedByCoordinate”. Alignment quality control (QC) and read mapping statistics were obtained from Picard v2.20.3 tools using function “CollectMultipleMetrics” [27]. FastQC v0.11.8 was used to perform QC checks on the raw sequencing data.

### SNP genotype validation

Variant genotypes per patient were first validated in DNA sequencing samples using IGV [28]. Each patient’s total depth and allele frequency were recorded and an empirical assessment of genotype was made. If 100% of reads supported an alternative variant in germline DNA sequencing then the patient was deemed homozygous for that particular variant. If < 100% and > 0% of germline reads supported the variant, the patient was deemed heterozygous. Otherwise, the patient was deemed homozygous for the reference allele. This process overrode variant caller germline categorizations where disagreement was present.

### RNA-Seq expression measurement

Each genotype was annotated with total RNA read depth and RNA variant allelic fraction (VAF). Bam-readcount [29] was used to measure the depth and VAF of alternate alleles at the locus of each respective variant for each patient.

### Population minor allele frequencies

GnomAD [30] was queried to determine the population minor allele frequency (MAF) of variants of interest. Population MAF was then broken into major ethnic groups as follows: European non-Finnish, African/African American, Latino/Admixed American, Ashkenazi Jewish, South Asian, and East Asian.

### Protein structure modeling

The variant protein sequence was deposited in the Baker lab folding algorithm [31]. The deduced model was presented by ChimeraX [32].

## Results

### NGS sequencing and patient study cohort characteristics

For optimal assessment of normal tissue function associated with RT toxicity, we build a next generation sequencing (NGS) database that included somatic tumor and paired normal tissue [33, 34]. NGS was performed using the same sequencing platform for all samples to maximize consistent data output for discovery of germline variants implicated in RT toxicity. Our dataset included 37 HNSCC patients (n = 37) with NGS data; 34 of which were HNSCC patients treated with curative intent and analyzed for clinical outcomes. Their characteristics are summarized in Table 1. Excluded patients include one with metastatic disease at diagnosis and two with locoregionally advanced cutaneous SCC. Metastatic disease was excluded due to inability to evaluate time to certain outcomes, such as metastasis free survival. Patients with cutaneous SCC were excluded due to differences in treatment paradigm, disease course and prognosis. While these patients were not evaluated for clinical outcomes they were retained for NGS analyses to assess for potential biomarkers associated with differing radiation AEs.

**Table 1:** Patient characteristics

Characteristics	Group A n = 16	Group B n = 18	Total n = 34^a^
	n (%)	n (%)	n (%)
Age at diagnosis			
< 65	10 (62.5)	8 (44.4)	18 (52.9)
≥ 65	6 (37.5)	10 (55.6)	16 (47.1)
Gender			
Male	15 (93.8)	16 (88.9)	31 (91.2)
Female	1 (6.2)	2 (11.1)	3 (8.8)
Smoking			
≤ 10 pack years	11 (68.8)	14 (77.8)	25 (73.5)
> 10 pack years	5 (31.2)	4 (22.2)	9 (26.5)
T stage			
0	1 (6.2)	0 (0.0)	1 (2.9)
1	3 (18.8)	3 (16.7)	6 (17.6)
2	5 (31.2)	5 (27.8)	10 (29.4)
3	4 (25.0)	3 (16.7)	7 (20.6)
4	3 (18.8)	7 (38.9)	10 (29.4)
N stage			
0	4 (25.0)	3 (16.7)	7 (20.6)
1	4 (25.0)	3 (16.7)	7 (20.6)
2	8 (50.0)	6 (33.3)	14 (41.2)
3	0 (0.0)	6 (33.3)	6 (17.6)
Overall stage			
I	3 (18.8)	2 (11.1)	5 (14.7)
II	1 (6.2)	2 (11.1)	3 (8.8)
III	2 (12.5)	2 (11.1)	4 (11.8)
IVa	8 (50.0)	6 (33.3)	14 (41.2)
IVb	2 (12.5)	6 (33.3)	8 (23.5)
p16 status			
Positive	5 (31.2)	8 (44.4)	13 (38.2)
Negative	7 (43.8)	9 (50.0)	16 (47.1)
Unknown	4 (25.0)	1 (5.6)	5 (14.7)
Primary site			
Oral cavity	4 (25.0)	6 (33.3)	10 (29.4)
Oropharynx	8 (50.0)	7 (38.9)	15 (44.1)
Larynx	2 (12.5)	3 (16.7)	5 (14.7)
Hypopharynx	0 (0.0)	1 (5.6)	1 (2.9)
Nasopharynx	1 (6.2)	0 (0.0)	1 (2.9)
Nasal cavity	0 (0.0)	1 (5.6)	1 (2.9)
Unknown	1 (6.2)	0 (0.0)	1 (2.9)
Primary treatment			
Definitive	12 (75.0)	8 (44.4)	20
Post-operative	4 (25.0)	10 (55.6)	14
Concurrent chemotherapy			
Yes	13 (81.2)	14 (77.8)	27 (79.4)
No	3 (18.8)	4 (22.2)	7 (20.6)

^a^3 patients were excluded from clinical analysis (one was treated palliatively and was metastatic at diagnosis, two had cutaneous SCC

### RT patient germline variants associated with increased late toxicity

There are previously reported findings in HNSCC pointing to differential tissue response in acute and late toxicity, where acute toxicity is associated with inflammation while necrotic developments may drive late toxicity outcomes [35]. To isolate contributing genetic factors in early and late responses, the patient cohort was divided into two groups (Group A and Group B) based on their toxicity profile. Specifically, for this grouping, patients in Group A had significant increase in late toxicity while patients in Group B did not experience a late stage change in toxicity. Based on these criteria 18 patients were designated as Group A and 19 patients were designated as Group B.

We tested for germline variant association with RT toxicity outcomes, restricting to germline variants that were present in at least 25% MAF with significant p-value ≤ 0.05 between Groups A and B. Among the top 30 germline variants for Groups A and B, we identified 5 that met our selection criteria: human leukocyte antigen (HLA-A), tuberous sclerosis complex 2 (TSC2), marker of proliferation Ki-67 (MKI67), interferon induced protein with tetratricopeptide repeats 2 (IFIT2), and interleukin 10 receptor subunit alpha (IL10RA).

### HLA-A Arg68Lys/Val91Met germline variant

We found 13 patients representing 37.1% of our cohort that carried the HLA-A Arg68Lys/Val91Met variant with 10 patients in Group A and 3 in Group B (p-value = 0.052, Table 2).

**Table 2:**
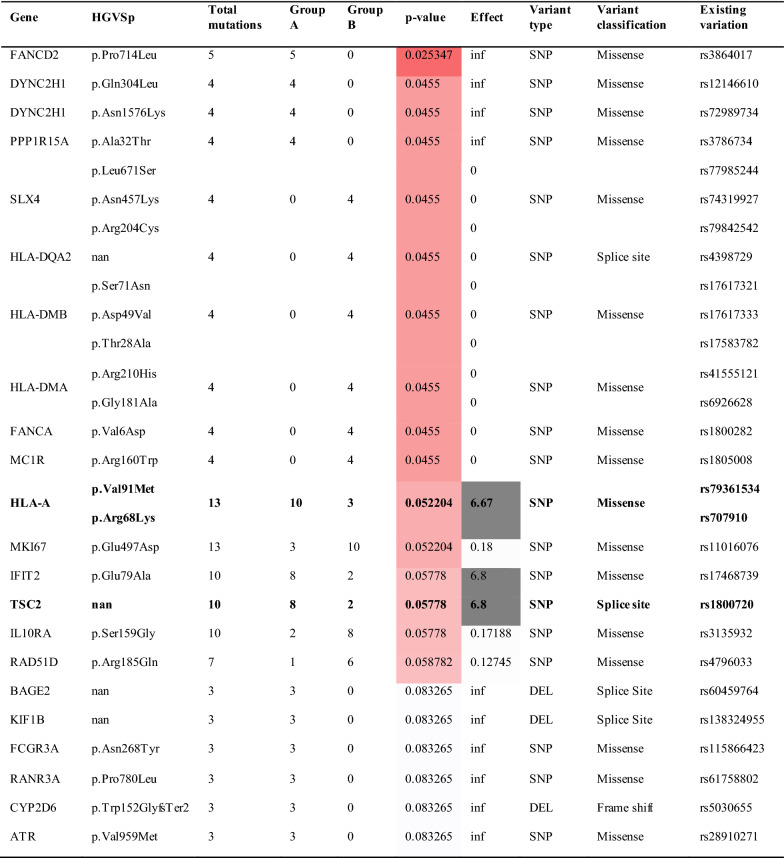
Chi-square results: recurrent freebayes mutations expanded beyond tumor suppressors and oncogenes

Patients in Group A experienced a higher CTCAE grade for late dysphagia compared to their early dysphagia. Patients in Group B experienced the same or lower CTCAE grade for early and late dysphagia. The effect value > 1 is the odds ratio for increased proportion of germline variants in Group A

**Fig. 1:**
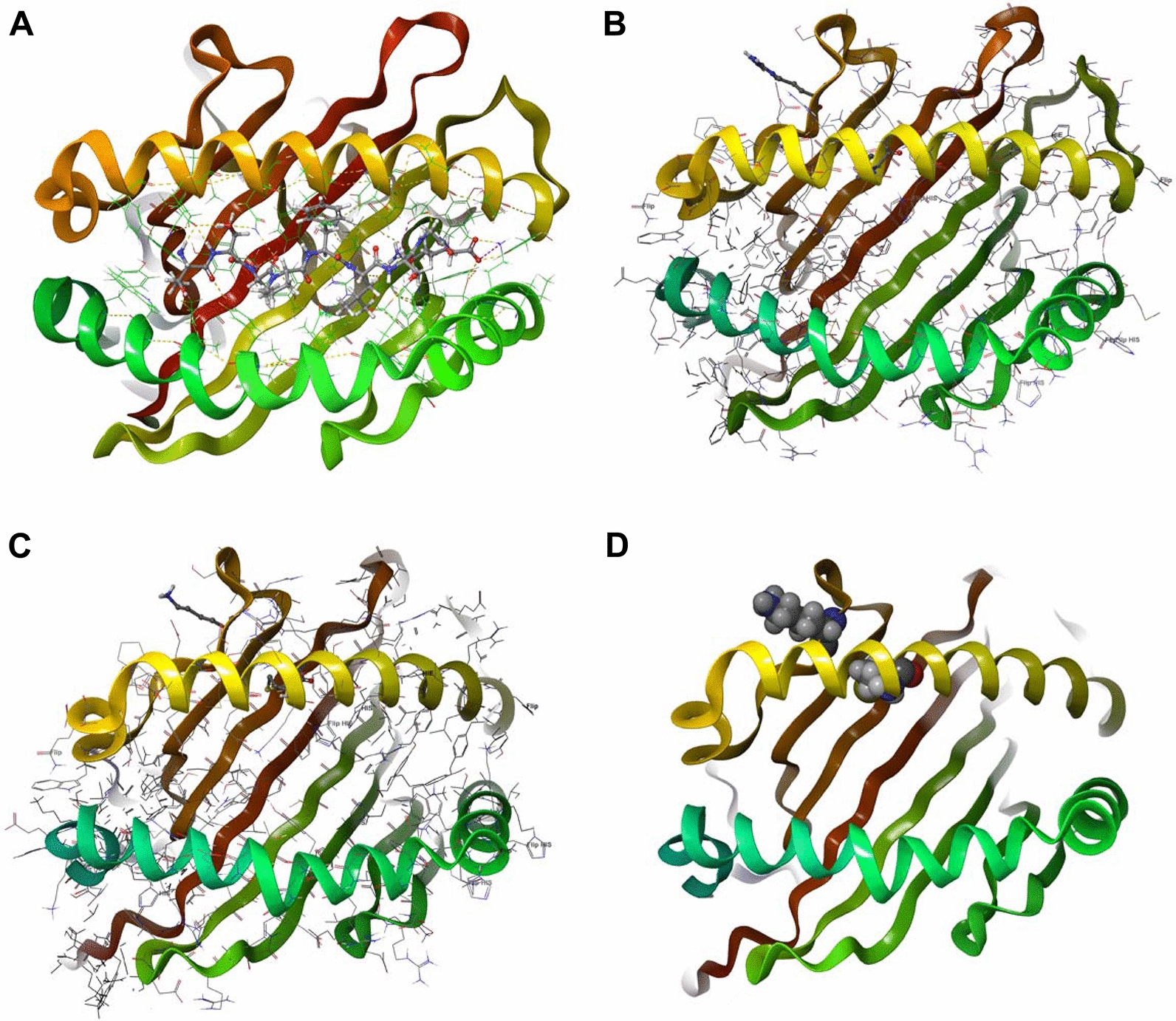
Structural HLA. **A** 3D structure of human HLA-A (609C) decorated by an epitope. **B** HLA-A wild type version (arginine to valine substitution). **C** HLA-A double mutant version (lysine to methionine substitution). **D** HLA-A double mutant version (lysine methionine) CPK presentation

All patients with the HLA-A Val91Met variant (rs79361534) also carried the HLA-A Arg68Lys variant (rs707910). This particular variant is documented in the HLA-A polymorphism database with a low MAF in a population of 1000 [36]. We found new characteristics of this HLA-A variant that have not been reported previously. This particular variant co-caries two isolated single nucleotide changes resulting in two amino acid substitutions at protein location 68 and 91. This has not been reported before since the two changes are listed as occurring separately and resulting in two HLA-A polymorphism genes (rs79361534 and rs707910). To examine the impact of these changes on the structure and function of the encoded protein, we performed a 3D modeling analysis (Fig. 1). We found that the valine substituted by methionine at position 91 is situated in the protein peptide binding groove consisting of two α-helixes and thus it is reasonable to conjecture that this amino acid substitution could be a factor in modified peptide binding affinity resulting in neo-antigen presentation. The Arginine substitution with Lysine at position 68 may introduce a new ubiquitin binding site, in addition, this variant was predicted as deleterious by using the Sorting Intolerant from Tolerant (SIFT) algorithm [37]. This HLA variant was not significantly associated with differences in PFS (p = 0.402), LRR (p = 0.173), MFS (p = 0.769) or OS (p = 0.757, Table 3). The effect size for this variant was high (6.67) signifying higher odds of late RT toxicity and potentially serving as a biomarker for RT stratification.

**Table 3:** P-values for PFS, LRR, MFS, and OS for patients with these variants vs. without

Variant	PFS	LRR	MFS	OS
TSC2	0.216	**0.018**	0.702	0.685
HLA-A V91M^a^	0.402	0.173	0.769	0.757
HLA-A R68K^a^				
FANCD2	**0.039**	0.774	**0.018**	0.448
IFIT2	0.163	0.127	0.852	0.202
GEN1	0.164	0.154	0.750	0.138
NCOR2	0.712	0.564	0.660	0.503
TET2	0.067*	0.306	0.266	0.495
MKI67	0.025	0.480	0.151	0.195
FANCI	0.189	0.910	0.376	0.687
RAD51D	0.590	0.654	0.807	0.280
IL10RA	0.340	0.084	0.600	0.972
IDH1	0.861	0.607	0.831	0.257
HLA-F N353L	0.266	0.901	0.046	0.262
FANCA V6D^b^	0.157	0.270	0.722	0.839
FANCA 5^c^	0.346	0.623	0.244	0.070
HLA-B Y140F	**0.043**	0.283	0.165	0.123
HLA-DMA/B 5^d^	0.456	0.041	0.966	0.213
DYNC2H1 Q304L	0.531	0.773	0.245	0.120
DYNC2H1 N1576K	0.231	0.511	0.926	0.243
PPP1R15A	**0.008**	**0.028**	0.191	0.255
SLX4	0.100	0.195	0.219	0.552
HLA-DQA2	0.507	0.308	0.662	0.887

P-values with bold text are < 0.05 with the variant conferring improved outcomes, those with underline text are associated with worse outcomes, and those marked with an asterisk are approaching significance

^a^These two variants appear together in the same group of patients

^b^Overlap with patients with MC1R R160W

^c^Group of patients with the same 5 changes in FANCA

^d^5 HLA mutations present in the same group (HLA-DMB S71N, D49V, T28A and HLA-DMA R210H, G181A)

### TSC2 splice variant

Our data analysis identified an intronic C>T TSC2 variant (rs1800720) flanking a splice donor site and occurring in 10 patients (28.6%) in our n37 cohort, with a trend towards a significant enrichment in Group A. The variant was detected in 8 Group A patients versus 2 in Group B (p = 0.0577, Table 2). This TSC2 variant was particularly enriched in our cohort (MAF = 0.189) relative to the gnomAD data (MAF = 0.0968) [30]. Interestingly, the MAF in our cohort closely aligns with the African/African American gnomAD population (MAF = 0.202).

This specific TSC2 splice region is identified as an associated germline variant in tuberous sclerosis syndrome. TSC2 in complex with TSC1 has tumor suppressor functions via regulation of the mechanistic target of rapamycin kinase (mTOR) signaling pathway [38]. Aberrant activation of the mTOR pathway has been widely implicated in HNSCC [39] and it is possible that this TSC2 variant is associated with HNSCC via effects on the mTOR pathway. Clinical outcome KM analysis for this variant is associated with lower risk of LRR (p = 0.018), however no association was found with PFS (p = 0.216), MFS (p = 0.702) and OS (p = 0.685, Table 3). This is the first time to our knowledge that the finding of this germline variant is reported in HNSCC.

### Other, low frequency germline variants in HNSCC

We detected 9 germline variants of significance associated with early versus late toxicity that did not meet our biomarker selection criteria due to less than 25% frequency of occurrence (Table 2). These were Fanconi anemia complementation group D2 (**FANCD2**) pPro714Leu (rs3864017); dynein cytoplasmic 2 heavy chain 1 (**DYNC2H1**) pGly304Leu (rs12146610; rs72989734); protein phosphatase 1 regulatory subunit 15A (**PPP1R15A**) pAla32Thr (rs3786734); SLX4 structure-specific endonuclease subunit (**SLX4**) pSer71Asn, pAsn457Lys, pArg204Cys (rs77985244, rs74319927, rs97842542); major histocompatibility complex, class II, DQ alpha 2 (**HLA-DQA2**) splice site (rs4398729); major histocompatibility complex, class II, DM beta (**HLA-DMB**) pSer71Asn, pAsp49Val, pThr28Ala (rs17617321, rs17617333, rs17583782); major histocompatibility complex, class II, DM alpha (**HLA-DMA**) pArg210His, pGly181Ala (rs41555121, rs6926628); Fanconi anemia complementation group A (**FANCA**) pVal6Asp, (rs1800282); and melanocortin 1 receptor (**MC1R**) pArg160Trp (rs1805008). The known functions of these genes cluster into two main functional families, 3 out of 8 are DNA repair genes associated with Fanconi anemia: FANCD2, SLX4, and FANCA [40–45], while 3 out the 8 are HLA genes comprising the major histocompatibility complex (MHC) involving immunity. The remaining genes are associated with skeletal neogenesis [46–48], protein synthesis homeostasis [49] and skin pigmentation [50]. All these germline variants were previously reported in the general population validating our findings, however their structure function-relationship in their individual encoded proteins awaits characterization. We report here that these gene variants occur in HNSCC patients and could be associated with late toxicity. Based on our study design, we found that FANCD2, DYNC2H1, PPP1R15A and HLA-A variants are associated with a significant increase in late toxicity, while SLX4, HLA-DQA2, HLA-DMB, HLA-DMA, FANCA, and MC1R variants are associated with fewer late RT toxicity events.

### Cumulative RT toxicity variants

In addition to early versus late toxicity gene variant effects, we analyzed cumulative toxicity associations, Group C (Grade 2–4) and Group D (Grade 0–1) in the same cohort of patients (n = 37, Table 4).

**Table 4:**
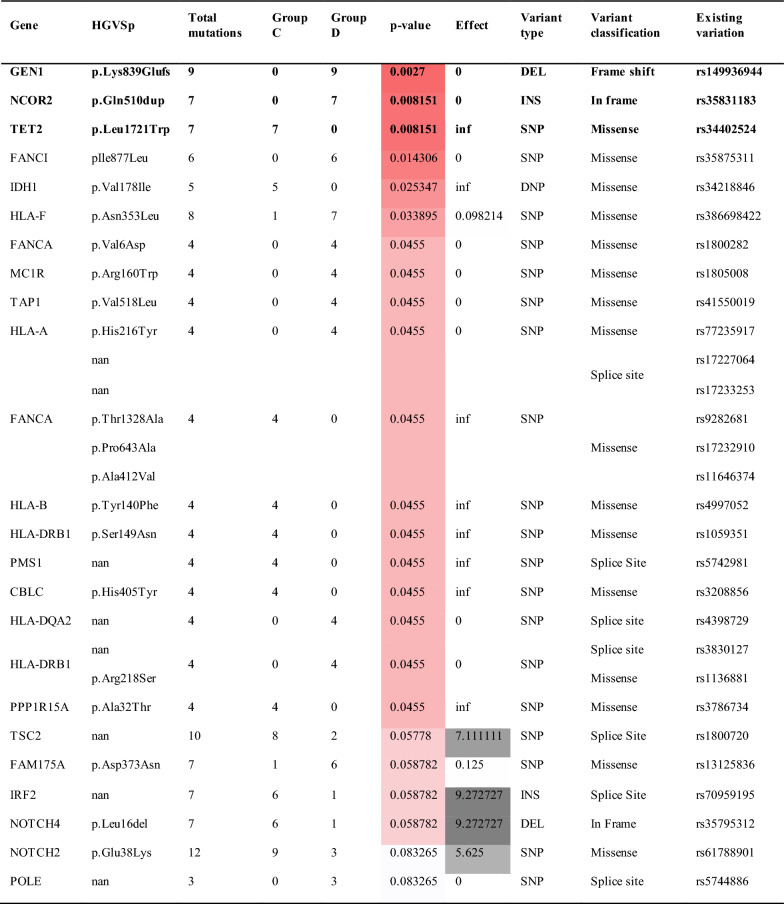
Chi-square results: recurrent freebayes mutations expanded beyond tumor suppressors and oncogenes

Patients in Group C experienced high toxicity for late dysphagia (CTCAE grade 2 +), while patients in Group D experienced a low toxicity for late dysphagia (CTCAE grade 0–1)

**Fig. 2:**
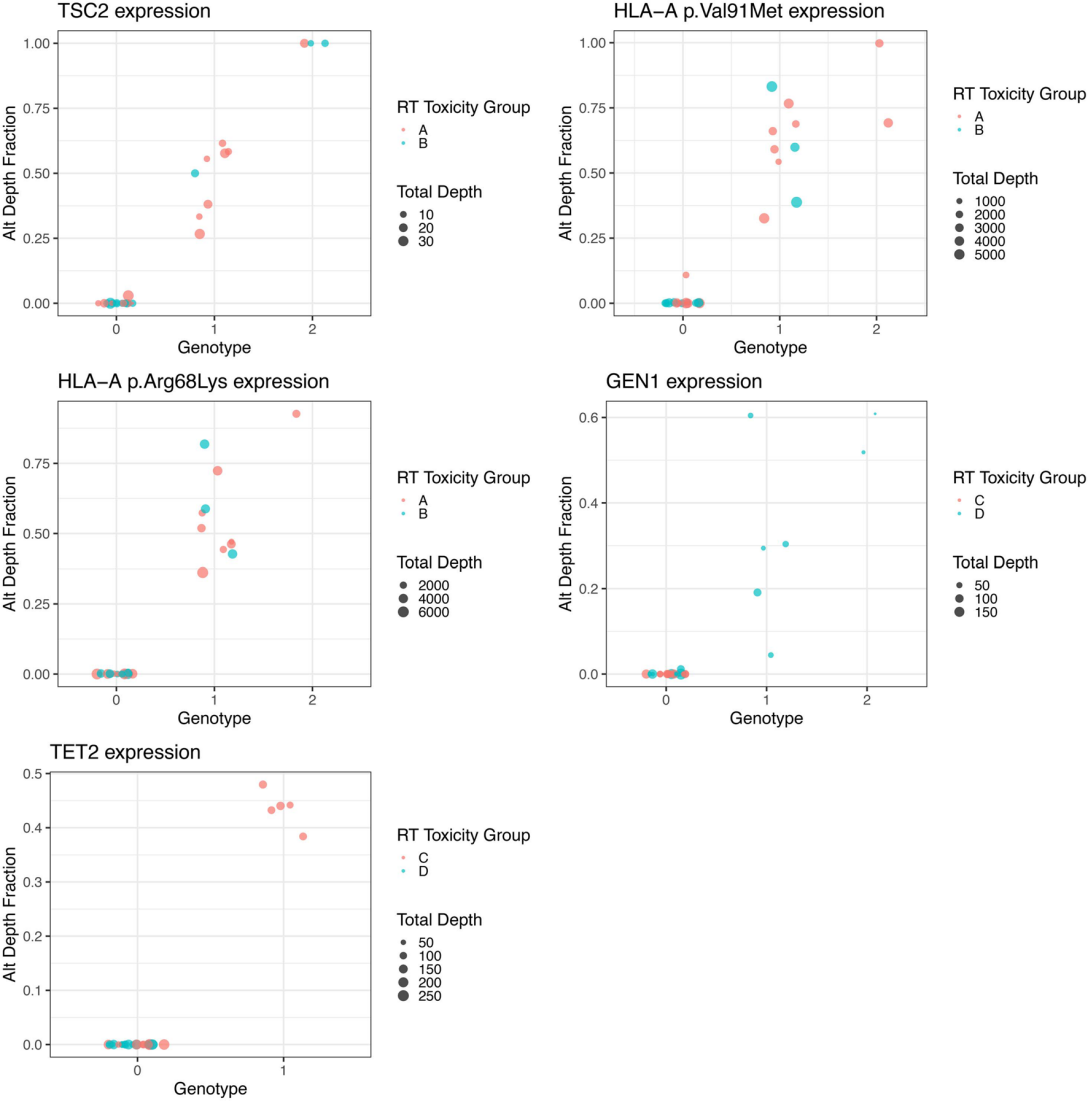
Bulk RNA-Seq derived variant allelic fraction (VAF) was generated for each variant and each patient. VAF values (y-axis) were compared against curated patient germline genotype values (x-axis) for each variant, where 0 = homozygous no variant, 1 = heterozygous variant, 2 = homozygous variant. Point color represents radiation toxicity group and point size represents total read depth at the variant locus of interest

Not surprisingly, the TSC2 splice site variant (rs1800720) detected in our previous study design displayed significant cumulative toxicity association. In addition, we detected a new variant GEN1, a Holliday junction 5′ flap endonuclease (GEN1) pLys839Glufs (rs149936944) that was associated with significant protection from RT therapy induced toxicity. We found that 9 patients carried this variant and that 2 of them were bi-allelic (Fig. 2). We confirmed this finding by analyzing the RNA-seq data. The reference GEN1 protein is 908 amino acids long. The variant we detected has a frame shift mutation introducing a stop codon at position 839 resulting in truncated messenger RNA (mRNA) missing 69 amino acids at the C-terminus of the GEN1 protein with potential for nonsense-mediated RNA decay. The presence of homozygosity (Fig. 2) of this variant implicates GEN1 redundancy, however, it is possible that this change leads to an alternate biological function. Indeed, GEN1 redundancy has been reported by Wang et.al [51]. They found that GEN1 and essential meiotic structure-specific endonuclease 1 (EME1) play redundant roles in meiotic recombination in a mouse model and that deletion of both genes confer synthetic lethality in mice [51]. In addition, a GEN1 knockout mouse is viable [52, 53].

We found that the nuclear receptor corepressor 2 (NCOR2) Gln510dup variant (rs35831183), showed a strong association with Group D (p = 0.008151).

### Clinical outcomes associated with selected germline variants

We also examined clinical outcomes between the two sets of groups and the implication of certain variants. For this portion of the analysis only those patients with HNSCC treated with definitive or post-op radiation/chemoRT were included. A total of 34 patients were included with radiation treatment dates ranging from January 2009 to January 2021. There were 14 patients (41.2%) that were treated in the post-operative setting. Radiation dose was available for 31 (91.2%) of patients, ranging 60–70 Gy (Gy). 27 patients (79.4%) received concurrent systemic therapy, most frequently cisplatin (70.4%). Median age at treatment was 62.5 years (range 37–85, Table 1), with a median follow up of 42.5 months (range 7–205). Between Groups A vs. B and Groups C vs. D there were no significant differences in age, stage, site of primary, HPV status, use of surgery or concurrent chemotherapy. Patient characteristics can be found in Table 1. 11.7% of patients had Grade 3 early mucositis or dysphagia and 29.4% of patients had Grade 3 dysphagia; no Grade 4 or 5 AEs were recorded.

**Fig. 3:**
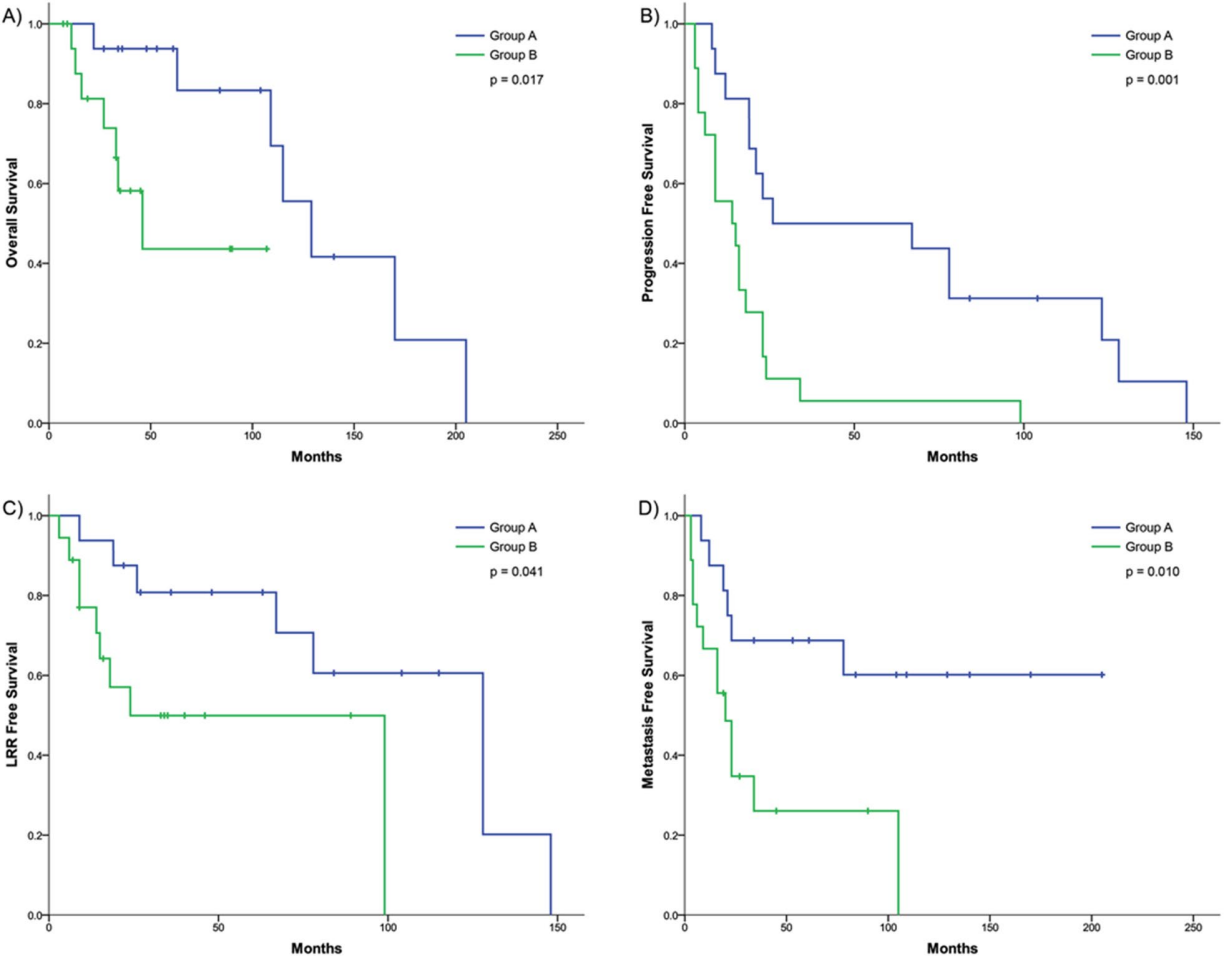
Analysis of outcomes for Groups A and B by **A** OS, **B** PFS, **C** LFS and **D** MFS. Log-rank p-values are shown

Patients in Group A had significantly better OS in comparison to Group B (p = 0.017, Fig. 3). There were also significant differences in PFS (p = 0.001), LFS (p = 0.041) and MFS (0.010). As noted previously, alterations in TSC2 were more prevalent in elevated late toxicity Group A, and patients with variants in TSC2 also had significantly improved LFS (p = 0.018, Table 3). FANCD2 was also more prevalent in Group A and associated with improved PFS (p = 0.039) and MFS (p = 0.018), as was PPP1R15A with PFS (p = 0.008) and LFS (0.028). There were five HLA-DMB/HLA-DMA variants only present in patients in Group B which were associated with a higher risk of LRR (p = 0.041). Within Groups C and D the major HLA-F N353L variant was significantly more prevalent in Group D and associated with more frequent distant metastases (p = 0.046).

## Discussion

We designed this pilot study to assess the viability of our method for detecting genetic variants associated with RT toxicity in HNSCC. In an earlier published study we found that somatic tumor mutations can be associated with RT toxicity [54]. In this study, we analyzed germline variants to identify biomarkers of RT toxicity that could aid patient stratification for personalizing radiation. Germline genetic biomarkers would be preferred for this purpose due to the ease of sample collection (blood/saliva) for targeted NGS testing.

While we did not aim to study the mechanism of action of these genetic variants, our findings pave the way to further the research deciphering the functional implications of these genes in HNSCC and other cancer types. Importantly, the majority of the variants we identified had several fold higher MAF in HNSCC patients than in the general population, which is suggestive of their potential role in HNSCC. Furthermore, the Fanconi anemia DNA repair genes were widely detected in our study. Since they appeared at a low frequency they did not meet our biomarker selection criteria. However, we found that these germline variants had strong, often opposite associations with RT toxicity outcomes. FANCD2 pPro714Leu and FANCA pVal6Asp correlated with increased toxicity while SLX4 variant comprising 3 different missense changes in this gene had protective effects in toxicity. The triple missense single nucleotide alteration resulted in amino acid changes Ser71Asn, Asp49Val, Thr28Ala suggestive of structural and functional selective pressures, and we are the first to report this finding. Another variant of interest is GEN1 and we verified that this variant transcript is expressed (Fig. 2), and thus may play a functional role in DNA repair. Although it is widely reported that DNA repair mechanisms play an important role in radiation sensitivity the protective effects of specific variants, such as the SLX4 triple variant, cannot be overlooked. Taken together, we observed a large number of DNA repair and MHC polymorphisms consistent with widely reported mechanistic studies of drivers of toxicity.

The clinical outcome data for all the germline variants selected for biomarker development was evaluated to isolate the significance of these variants for clinical outcomes in order to ensure that utilizing these biomarkers in adapting the radiation dose would not diminish treatment benefits. Specifically, GEN1, HLA-A and TSC2 germline variants meet this criteria for biomarker development. All carriers of GEN1 frame shift variant pLys839Glu exhibited significantly lower cumulative toxicity (p = 0.027) as determined by CTCAE grading. We did not detect significant correlation with clinical outcomes for this variant (Table 4). The HLA-A variant was associated with late toxicity in 10 of 13 patients (p = 0.052). The presence of this variant did not affect clinical outcomes while it was found in 35% of our cohort. We consider this variant as a good biomarker candidate.

Other gene variants we identified present a rationale for new therapeutic targets in HNSCC. In particular, the TSC2 splice site variant found in 27% of patients was significantly associated with cumulative as well as late stage toxicity. This particular variant (rs1800720) is implicated in Tuberous Sclerosis syndrome. TSC2 in complex with TSC1 has a regulatory role in the mTOR signaling pathway in HNSCC [38]. We detected the variant transcript expression as heterozygous in 8 patients and homozygous in 2 patients (Fig. 2). Characterizing this variant in experimental models may further aid in decoding the TSC2/mTOR function in HNSCC. Other variants we identified, while occurring at a lower frequency, can further aid in investigating their functional implications, thus shedding light on their “normal” function in HNSCC. An additional notable result of our study which deserves further investigation is the finding that patients exhibiting higher RT toxicities show significant beneficial clinical outcomes. A similar pattern has been observed in patients with ATM mutations, where it has been suggested as a potential biomarker for rtAEs with several studies also showing it conferred improved treatment response to radiation [55–57].

There are limitations to our study including the limited sample size and retrospective nature. Based on this pilot data we are designing a larger study with prospective validation cohorts to further investigate these findings.

## Conclusions

In summary we identified unique germline variants which were associated with various patient outcomes including long term toxicity, LRR, and OS after radiation therapy in HNSCC. These findings provide rationale for larger studies incorporating genetic biomarkers to better predict responses to radiation therapy and advance personalized radiation medicine.

## Data Availability

The datasets used, generated, and analyzed during the current study are available from the corresponding author on reasonable request.

## Abbreviations

AJCC: American Joint Committee on Cancer
ATM: ATM serine/threonine kinase
CTCAE: Common Terminology Criteria for Adverse Events
DNA: Deoxyribonucleic acid
DYNC2H1: Dynein cytoplasmic 2 heavy chain 1
EME1: Essential meiotic structure-specific endonuclease 1
FANCA: Fanconi anemia complementation group A
FANCD2: Fanconi anemia complementation group D2
GEN1: GEN1 Holliday junction 5′ flap endonuclease
Gy: Gray
HLA-A: Major histocompatibility complex, class I, A
HLA-DMA: Major histocompatibility complex, class II, DM alpha
HLA-DMB: Major histocompatibility complex, class II, DM beta
HLA-DQA2: Major histocompatibility complex, class II, DQ alpha 2
HNC: Head and neck cancer
HNSCC: Head and neck squamous cell carcinoma
HPV: Human papillomavirus
IFIT2: Interferon induced protein with tetratricopeptide repeats 2
IL10RA: Interleukin 10 receptor subunit alpha
IMRT: Intensity modulated RT
KM: Kaplan–Meier
LFS: Locoregional failure free survival
LRR: Locoregional recurrence
MAF: Minor allele frequency
MC1R: Melanocortin 1 receptor
MFS: Metastasis free survival
MHC: Major histocompatibility complex
MKI67: Marker of proliferation Ki-67
mRNA: Messenger RNA
mTOR: Mechanistic target of rapamycin kinase
NCOR2: Nuclear receptor corepressor 2
NGS: Next generation sequencing
NSCLC: Non-small cell lung cancer
OAR: Organs at risk
OS: Overall survival
PFS: Progression free survival
PPP1R15A: Protein phosphatase 1 regulatory subunit 15A
QC: Quality control
RNA: Ribonucleic acid
RNA-seq: RNA-sequencing
RT: Radiation therapy
rtAE: RT adverse event
SEER: Surveillance, epidemiology, and end results
SIFT: Sorting Intolerant from Tolerant
SLX4: SLX4 structure-specific endonuclease subunit
TET2: Tet methylcytosine dioxygenase 2
TSC2: TSC complex subunit 2
VAF: Variant allelic fraction
